# Preliminary Findings of a Chronic Disease Management Program in Medicare Advantage Enrollees with Mild to Moderate Kidney Disease

**DOI:** 10.3390/ijerph23020237

**Published:** 2026-02-13

**Authors:** Trevon Morales, Rubette Harford, Dulcie Kermah, Jose Flaque, Michelle Camacho, Damaris Vasquez, Vanessa Schmidt, Inés Hernández-Roses, James P. O’Drobinak, Keith C. Norris

**Affiliations:** 1 David Geffen School of Medicine, University of California, Los Angeles, CA 90095, USA; trevonmorales@mednet.ucla.edu (T.M.); vanessa.arguello@gmail.com (V.S.); 2 Atlantis HealthCare Inc., Trujillo Alto, PR 00978, USA; rhrenalhealth@gmail.com (R.H.); drjoseflaque@gmail.com (J.F.); michelle.camacho@atlantishgi.com (M.C.); damaris.vazquez@atlantishgi.com (D.V.); 3 School of Medicine, Charles R Drew University of Medicine and Science, Los Angeles, CA 90059, USA; 4 MCS Healthcare Holdings, LLC, San Juan, PR 00916-1919, USA; irhernandez@medicalcardsystem.com (I.H.-R.); jim.odrobinak@medicalcardsystem.com (J.P.O.)

**Keywords:** CKD, diabetes, prediabetes, Puerto Rico, chronic disease management

## Abstract

**Highlights:**

**Public health relevance—How does this work relate to a public health issue?**

- Chronic kidney disease (CKD) represents a major public health issue in Puerto Rico, where prevalence and progression rates exceed those of the continental United States. This is largely impacted by high diabetes prevalence, structural healthcare inequities, and limited access to specialty care.
- Our work directly addresses the public health challenge of CKD progression by evaluating a large-scale multidisciplinary chronic disease management program designed to improve early detection, care coordination, and disease stabilization in an under-resourced population.

**Public health significance—Why is this work of significance to public health?**

- By demonstrating that over 90% of real-world patients with CKD stages 2–3 experienced stabilization or improvement in renal function, our work provides evidence that early, coordinated interventions can meaningfully alter CKD trajectories at the population level.
- Our findings are particularly significant for public health because they highlight an effective care model within Puerto Rico’s complex Medicare Advantage and health system barriers, addressing a population historically underrepresented in CKD research.

**Public health implications—What are the key implications or messages for practitioners, policy makers and/or researchers in public health?**

- For practitioners, the results support early nephrology engagement and multidisciplinary care as critical strategies to prevent CKD progression, reduce possible dialysis burden, and improve outcomes even in high-risk populations like the elderly.
- For policymakers and researchers, our study provides preliminary evidence to expand and sustain Medicare Advantage based chronic disease management programs, even in structurally disadvantaged settings. Longer term evaluation on outcomes on morbidity, mortality, and cost-effectiveness will hopefully further support investing in a multidisciplinary care model for patients with early-stage CKD.

**Abstract:**

Background: Chronic kidney disease (CKD) is traditionally viewed as a condition marked by a progressive reduction in kidney function leading to the need for kidney dialysis or transplantation. The estimated prevalence of CKD in adults in Puerto Rico is ~20% higher than that of the overall United States (US). To address the disproportionately high rate of CKD in Puerto Rico, we created a multidisciplinary chronic disease management (CDM) program targeting CKD and diabetes mellitus (DM), the leading CKD risk factor. Methods: Over 7200 eligible enrollees in a Puerto Rico-Managed Medicare Program participated in a CDM program targeting individuals with CKD or DM as determined by administrative review. Evaluations were conducted on 4068 program participants with baseline glomerular filtration rate (eGFR) and codifying CKD stage by eGFR. A dietitian/nurse team provided dietary and lifestyle recommendations to the patient/family and a nephrologist/endocrinologist made diabetes and CKD recommendations to the primary care provider. Findings on 2095 participants with Stages 1–3 CKD with follow-up eGFR at least 6 months but less than 2 years after baseline are presented. Results: At baseline, the mean age was 74 years (range 30–101), 59% of patients were female and mean duration of follow-up from initial evaluation to second evaluation was 407 days (±159 days SD). Most participants had Stage 2 CKD (34.8%), followed by CKD Stage 1 and 3 (33.5 and 31.7%). During the follow-up period, 55.9% of participants with Stage 1 CKD remained in Stage 1, 84.9% of patients with Stage 2 remained in Stage 2 or regressed to Stage 1, while 96.1% of patients with Stage 3 remained in Stage 3 or regressed to Stage 2. Only 15.1% of patients in Stage 2 progressed to Stage 3 and 3.9% of patients in Stage 3 progressed to Stage 4 or 5. A secondary analysis comparing all 665 CDM Stage 3 participants to 117,249 historical controls found CDM participants demonstrated a higher rate of regression (20.3% vs. 15.2%; absolute difference +5.1 percentage points; *p* = <0.01) and a lower rate of progression (3.9% vs. 15.3%; absolute difference −11.4 percentage points; *p* < 0.001). Conclusions: Early findings of a multidisciplinary CDM intervention indicate that 79% of participants with CKD Stages 1–3 by eGFR had stabilized or improved CKD status. Comparison to a randomized control group to better assess for causality and longer-term CDM program follow-up on CKD status and clinical outcomes is warranted.

## 1. Introduction

Chronic kidney disease (CKD) is a condition characterized by a progressive decrease in kidney function, most typically documented by reduced estimated glomerular filtration rate (eGFR) or other indicators of renal injury such as albuminuria, a protein that is detectable in the urine of individuals with kidney injury [1]. CKD is prevalent in an estimated 37 million adults in the United States (US) and is linked with premature morbidity and mortality, as well as high healthcare expenses—particularly if the disease progresses to end-stage kidney disease (ESKD) requiring dialysis or a kidney transplant [2]. CKD is frequently caused or accompanied by other chronic illnesses such as diabetes and hypertension, and is thus a clinical and population health priority [3].

It is estimated that 16.8% of the adult population in Puerto Rico have CKD, 1.2-fold higher than the estimated 14% of adults with CKD in the US overall [4]. Diabetes is now the leading cause of CKD in the United States (US), and globally, and while it is estimated to account for as many as 40% of cases overall in the US [5], it is estimated to account for 66% of cases in Puerto Rico [6]. The high rate of CKD in Puerto Rico compared to the 50 US states appears to be multifactorial and may be driven not only by DM but differences in lifestyle, lower levels of health-affirming resources, inconsistent healthcare landscapes, fewer physician specialists and more [7,8]. These factors likely also underlie the high rate of ESKD in the territory, as Puerto Rico has more patients receiving dialysis treatments than at least 25 US states [6].

Diamantidis et al. noted an increasing prevalence of CKD among Medicare Advantage beneficiaries over the last 20 years, with Black and Hispanic patients having a more rapid decline in kidney function than their peers after enrollment, suggesting a need for closer surveillance and management to reduce their particular risk of faster progression [9]. Rivera-Hernandez et al. reported worse performance measure outcomes including diabetes and CKD among Hispanic residents in Puerto Rico enrolled in Medicare Advantage programs compared to Hispanic enrollees in the continental US [10]. These included 5 of 7 measures for comprehensive diabetes mellitus care (including hemoglobin A1c testing, hemoglobin A1c control, retinal eye examination, low-density lipoprotein cholesterol control, nephropathy screening), and 3 of 4 for cardiovascular care measures (including low-density lipoprotein control after coronary event, blood pressure control, and use of a β-blocker after myocardial infarction) [10].

## 2. Materials and Methods

**Figure 1:**
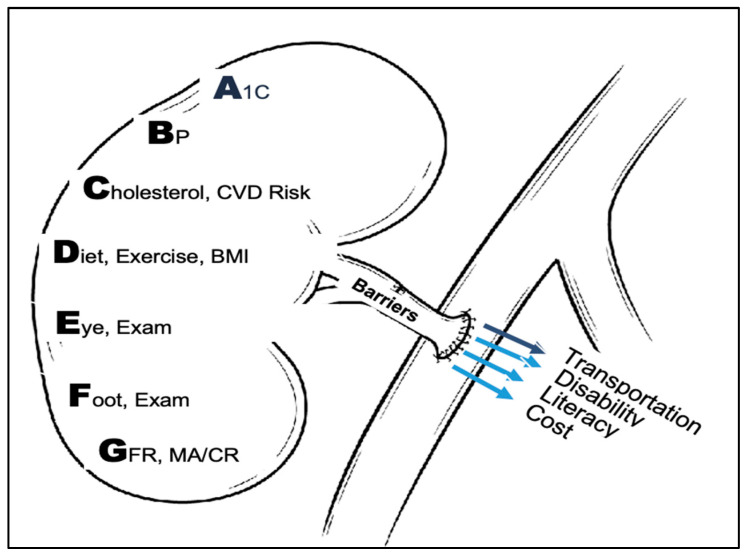
Key elements of the multidisciplinary chronic disease management program in pictorial form used in patient education. A1C—Hb A1C; BP—blood pressure; CVD—cardiovascular disease; BMI—body mass index; GFR—glomerular filtration rate; MA/CR—urine albumin to creatinine ratio including microalbuminuria.

To help meet the urgent need for early high-quality CKD care for Medicare Advantage enrollees in Puerto Rico, we created a multidisciplinary (dietitians, nurses, endocrinologist and nephrologist) chronic disease management (CDM) program for adult patients with or at risk for CKD (with an emphasis on diabetes as the primary risk factor) (Figure 1). The CDM program included the implementation of dietitian- and nurse-based patient and family education, lifestyle interventions and health-related social needs using a shared decision-making approach. The CDM program was used as an opportunity to elevate patient awareness about CKD risk factor control and complications of developing CKD, as well as to enhance coordination of follow-up care. This was done during the first year of patient assignment to the CDM program. Chart review was performed by a nephrologist and/or endocrinologist for possible recommendations to primary care physicians. Continuous monitoring of participant clinical responses to interventions and/or recommendations was conducted, with the development of new action plans as needed. We posited many enrollees with CKD Stages 1–3 based on estimated glomerular filtration rate (eGFR) who were enrolled in the CDM program and followed up between 6 months and 2 years would have low levels of CKD progression and some enrollees with Stage 2–3 CKD might even have regression to a better CKD stage.

### 2.1. Study Conduct

This study was conducted in accordance with the Declaration of Helsinki [11]. Informed consent was waived because the study only comprised secondary analysis of deidentified data, in accordance with 45 CFR §46. The program enrolled residents in Puerto Rico who were a part of MCS Healthcare Holdings, which offers a wide variety of Medicare Advantage plans under a contract with the Centers for Medicare & Medicaid Services (CMS). Deidentified participant demographic information included age, gender, city, and the Independent Physician Association (IPA) that was the provider of care. Other data included participants’ number of clinical encounters, interventions, date of interventions, and diagnosis.

The CDM program targeted MCS adult enrollees at least 18 years of age with diabetes mellitus or CKD Stages 1–3. We focused on patients with CKD for this analysis.

### 2.2. Measures and CKD Staging

CKD stage was determined by ICD-10 administrative code: N18.1—CKD Stage 1, N18.2—CKD Stage 2, and N18.3—CKD Stage 3. Nephrology assessment as per the 2021 CKD-EPI equation [12] was used to calculate estimated glomerular filtration rate (eGFR) and codify CKD stage. CKD stages by eGFR were defined using the Kidney Disease: Improving Global Outcomes (KDIGO) guidelines as follows [1,13,14]:

- CKD Stage 1: eGFR ≥ 90 mL/min/1.73 m^2^;
- CKD Stage 2: eGFR 60–89 mL/min/1.73 m^2^;
- CKD Stage 3: eGFR < 60 mL/min/1.73 m^2^.

We chose not to use albuminuria as part of the CKD definition, given that data were available for less than 15% of the entire cohort and we wanted to maintain consistency in the CKD definition.

The status of follow-up CKD stage from baseline was defined as follows:

- Progression: individual moving from Stage 1 to Stage 2 or 3, Stage 2 to Stage 3 or 4/5, and Stage 3 to Stage 4/5, based on follow-up eGFR.
- Stable or Regression: CKD stage remaining the same or improving to an earlier stage such as from Stage 2 to Stage 1 or Stage 3 to Stage 2 based on follow-up eGFR.

### 2.3. Covariates

Clinical factors, included a priori based on known relevance to CKD progression, were body mass index (BMI—kg/m^2^) and systolic and diastolic blood pressures (mmHg), which were collected as part of clinical visits. Hemoglobin A1c (HbA1c—%) and low-density lipoprotein (LDL—mg/dL) were reported by a local laboratory. We also assessed the use of common nephroprotective medications such as angiotensin-converting enzyme inhibitor (ACEi), angiotensin receptor blocker (ARB), sodium–glucose co-transporter (SGLT2) and glucagon-like peptide (GLP1). Similar to other electronic health record (EHR) real-world databases [15,16], urinary albumin/protein to creatinine ratios were available in only 14.6% of participants and therefore were not included in analyses. Thus, to maintain a consistency for CKD staging, we classified CKD stage by eGFR only.

Study participants eligible for this analysis were derived from the overall CDM program and included 7293 patients with or at risk for CKD, who were enrolled in the program as of September 2024, regardless of when they started the program. Of the enrolled participants, 4068 patients were evaluated based on baseline eGFR data, and were classified into CKD groups. Patients who were not eligible to be a part of the final analytical cohort included those who had missing follow-up data on eGFR, missing data on age and gender, less than 6 months of follow-up data, more than 2 years of service between the first and second eGFR assessment dates, or CKD Stage 4 or 5 at baseline.

Due to the high prevalence of complications, patients with CKD Stage 4 or 5 were beyond the scope of the CDM program and were referred to active nephrology care, including preparation for possible renal replacement therapy.

**Figure 2:**
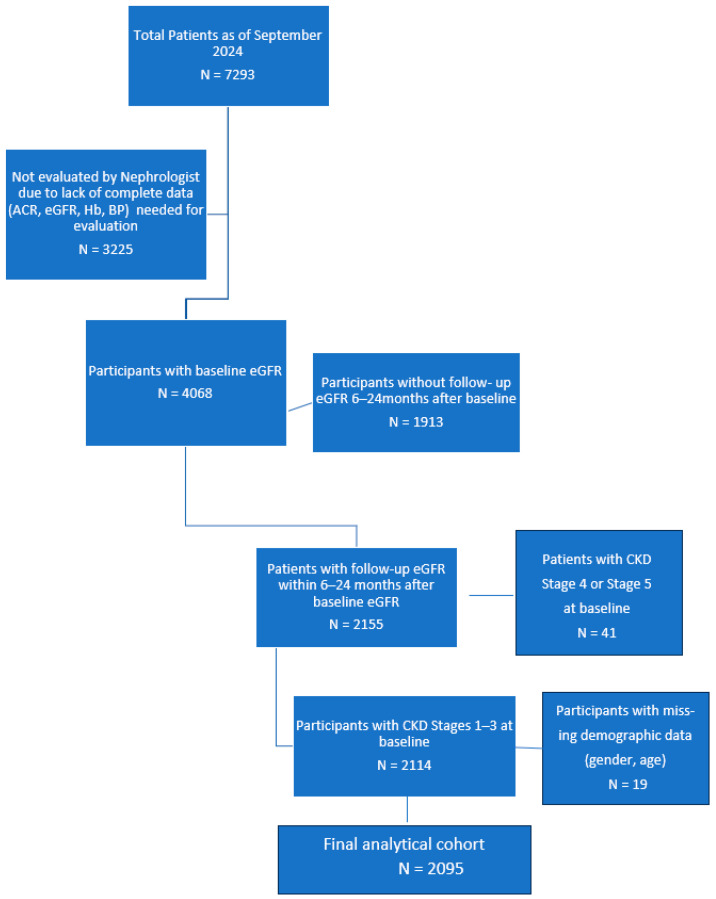
Consort diagram of algorithm used to define study cohort.

The final analytical sample was 2095 participants with CKD Stage 1–3 (Figure 2). This includes ~5% of participants with missing values for diabetes and hypertension. Descriptive analysis, including frequencies, percentages, and mean statistics, was calculated. Data were analyzed using SAS software V.9.4 (SAS Institute, Cary, NC, USA). Logistic regression analysis was performed to assess possible factors contributing to eGFR-based CKD regression or progression. Variables included age, gender, BMI, LDL, HbA1c <,≥ 7%, SBP <,≥ 140 mmHg, use of ACEi, ARBs and SGLT2/GLP1. A secondary analysis was conducted to compare CDM outcomes to historical benchmarks using one-sample proportion tests. Observed proportions and 95% confidence intervals were calculated. Statistical significance was set at *p* < 0.05.

## 3. Results

Among the 2095 participants in our final analytic cohort, 59% were female; 41% were male. The average age was 74 years old with an age range between 30 and 101 years. Most participants, 401 (39%), were between 65 and 74 years. The mean duration of follow-up from initial evaluation to second evaluation was 407 days (±159 days SD). ACEi, ARB, or SGLT2/GLP1usage was 1%, 6.9% and 7.2% respectively (Table 1). Participant distribution by CKD stage was 701 (33.5%) with Stage 1 CKD, 729 (34.8) with Stage 2 CKD, and 665 (31.7) with Stage 3 CKD (Table 1).

Clinical and laboratory values were similar at baseline among enrollees whose CKD stage remained stable/regressed and those whose CKD Stage regressed (Table 2). The use of ACEi/ARB was 7.4% among participants whose CKD Stage remained stable/regressed compared to 10.2% among those whose CKD Stage progressed. The use of SGLT2/GLP1was 7.3% among participants whose CKD Stage remained stable or regressed compared to a use of 6.7% among those whose CKD Stage progressed (Table 2).

During the follow-up period, 55.9% of participants with Stage 1 CKD remained in Stage 1, while 84.9% of patients with Stage 2 remained in Stage 2 or regressed to Stage 1, with a similar degree of enrollees regressing (16.2%) or progressing (15.1%). Overall, 96.1% of patients with Stage 3 were stable/regressed, and 20.3% regressed to a lower stage, more than five-fold the 3.9% who progressed to a worse CKD stage (Table 3).

Multivariable logistic regression analyses were conducted, including age, gender, BMI, LDL, HbA1c <,≥ 7%, SBP <,≥ 140 mmHg, use of SGLT inhibitors, ACEi, ARBs, and follow-up time. None of the covariates were significantly associated with eGFR-based CKD progression. ARB use was associated with a trend toward higher odds of progression (OR 1.52, 95% CI 0.97–2.37), although this did not reach statistical significance (*p* = 0.06).

Lastly, we conducted a secondary analysis comparing CKD Stage 3 outcomes with a historical cohort of 117,249 CKD Stage 3 patients in Alberta Canada that reported a regression rate of 15.2% and a progression rate of 15.3% [17]. A total of 665 patients with CKD Stage 3 from the CDM cohort were included. During the follow-up period, 135 patients (20.3%) regressed to a less severe CKD stage, 26 patients (3.9%) progressed to a more severe stage, and 504 patients (75.8%) remained at CKD Stage 3. Compared with the historical cohort, CDM participants demonstrated a higher rate of regression (20.3% vs. 15.2%; absolute difference +5.1 percentage points; *p* ≤ 0.01) and a lower rate of progression (3.9% vs. 15.3%; absolute difference −11.4 percentage points; *p* < 0.001).

## 4. Discussion

We found that the vast majority of patients with eGFR-defined CKD Stage 1–3, enrolled in a CDM program in Puerto Rico and followed from 6 months to 2 years, either remained at the baseline CKD stage or regressed to a lower stage. Among the subsets or enrollees with CKD Stage 2 or 3 at baseline, 84.9% and 96.1%, respectively, had their CKD stage remain stable or improve to a less severe stage. This is consistent with reports noting that while CKD has traditionally been considered a relentlessly progressive illness, more recent evidence indicates a more dynamic trajectory, with not only progression but also stabilization and even regression of kidney function now being possible [17,18,19]. Several of our findings were consistent with Liu and colleagues, who observed that among older adults in Alberta, Canada, with Stage 3 CKD, 15.2% of the cohort experienced progression (>25% fall in eGFR) and 15.3% experienced regression (>25% rise in eGFR) over the study period [17]. By contrast, for our enrollees at greatest risk, the proportion of patients with Stage 3 CKD who had regression of their CKD stage was five-fold higher than that of those with progression. In sum, such evidence supports emerging data that challenges traditional thinking about the relentlessly progressive nature of CKD and underscores the importance of early diagnosis, risk assessment and urgent intervention [18,19].

Our findings likely reflect the impact of targeted, patient-centered interventions that prioritize early detection supported by a multidisciplinary care team with early engagement, coordinated follow-up, and shared decision-making [20]. Diamantidis et al. also reported that kidney function declined more slowly in Medicare Advantage enrollees with clinical recognition of CKD [9]. This suggests that CKD progression can be attenuated when there is clear recognition of the disease and subsequent action taken. Early engagement with nephrologists can help diagnose more patients with CKD and allow for the initiation of timely care. The failure to detect patients before early intervention can lead to lost opportunities for prompt medical interventions. The direct patient management approach of the CDM program may have contributed to the high number of stable and regressive outcomes documented in our research population.

The need for a CDM program in Puerto Rico has been highlighted by several reports. Racial and ethnic minority Medicare Advantage beneficiaries in the 50 US states are disproportionately being enrolled in lower-performing plans with reduced nephrology services and care coordination assistance [21,22]. Specific to Puerto Rico, an analysis by Rivera-Hernandez and colleagues found that Hispanic Medicare Advantage enrollees in Puerto Rico showed substantially lower performance on 15 of the 17 performance measures examined than did Hispanic MA enrollees in the United States, including measures for comprehensive diabetes and cardiovascular care, such as CKD and hypertension [10]. In addition, for patients with CKD in Puerto Rico, it is important to note its health context and health systems operate on entirely different structural and political factors [8]. This is due to the issue of Puerto Rico still having a colonial relationship with the United States that leads to a paucity of economic control, high rates of poverty (45%) and unemployment (12%), and limited federal funding, with federal payment for MA programs and physician Medicare reimbursement being about 40% lower than average US state rates [7]. The inequitable federal Medicare and Medicaid support contributes to compromised quality of care with extensive wait times, limited appointment availability, the need to travel longer distances to receive care and a larger medication “doughnut hole” (until 2024), leading to larger out of pocket costs or lack of medications [8,23,24], creating a significant financial burden for many patients with CKD, especially those treated with dialysis or kidney transplants [24]. The confluence of these factors has led to shortages of specialists in Puerto Rico, as many physicians emigrate from the island to the US states to gain improved prospects, such as improved compensation and working conditions [8,25]. These realities reinforce the need for effective, targeted disease management programs to improve care.

Our study brings new insights by focusing on patients in Puerto Rico who are underrepresented in much of the CKD literature [26,27]. Most CKD outcome studies in the United States concentrating on Hispanic patients focus on Mexican Americans, with little disaggregated data available, thus failing to account for unique social and environmental risk profiles in Hispanic subgroups like Puerto Ricans and others [26,27]. In a cross-sectional study of over 15,000 U.S. Hispanic/Latino adults of Cuban, Dominican, Mexican, Puerto Rican, Central American and South American backgrounds enrolled in the Hispanic Community Health Study/Study of Latinos, Ricardo et al. reported that CKD prevalence varied across groups by nearly 50% for men and over 100% for women, with both men and women from Puerto Rico having the highest prevalence of CKD [27].

Prior critiques of the Medicare Advantage scheme indicated racial and ethnic minority Medicare Advantage participants were disproportionately placed in plans with less adequate benefits, a potential factor contributing to them suffering worse outcomes [21,22]. In addition, it has been reported that Medicare Advantage enrollees with accelerated eGFR decline have disproportionately higher costs than enrollees with mildly reduced kidney function [28]. To these ends, the MCS plan in Puerto Rico implemented a CDM program to ensure high-quality benefits to their high-risk enrollees, those with CKD and DM.

Although our study is valuable for providing insights, some limitations need to be noted. One is that the lack of a randomized control group precludes making causal inferences. Secondly, our population is confined to a particular geographic and insurance context and may limit generalizability to other groups of people. Thirdly, we were unable to control for many known and unknown confounders, including urinary albumin or protein excretion, due to limited data. Interestingly, a new report suggests SGLT2 inhibitor efficacy may be independent of urinary albumin excretion [29]. Fourth, the use of nephroprotective medications was low, only being used by <20% of patients, and could reflect low usage or underreporting in the EHR. Future analyses may require natural language processing to capture nurse and provider notes for more complete medication ascertainment. Fifth, selection bias based on participants being excluded from the study because of absent nephrologist assessments or missing data such as albuminuria can further limit generalizability. Lastly, long-term outcomes of hospitalization rates, mortality, and cost effectiveness were not measured but will be important areas to investigate further in the future. Even with these limitations, including a lack of causal evidence, the CDM program has provided a hypothesis, suggesting that a structured, multidisciplinary chronic disease management program may achieve important short-term gains in patient outcomes, even in a relatively under-resourced US healthcare setting.

What is impactful in this multi-disciplinary model is that it involves a patient- and family-centered approach with dietitians and nurses, supported by nephrology and endocrinology recommendations to primary care providers with tailored follow-up according to CKD stage. Such a design not only assists in helping CKD patients with their own care management but also reduces the burden on primary care physicians who experience high loads of patients to care for, as well as clinical guidelines from various medical societies to adhere to and increasing administrative tasks to perform [20]. In Puerto Rico, increased patient loads for primary care physicians are compounded by not only outmigration but an aging primary care physician population with a median age of 60 years compared to 48 years in the US overall, as well as low retention of local medical school graduates on the island [30]. Such increasing patient loads can make it difficult to not only detect CKD early, but to initiate early intervention and monitoring, further emphasizing the need for effective CDM programs.

## 5. Conclusions

To our knowledge, this study presents the first large-scale assessment of Medicare Advantage CKD management outcomes for adults in Puerto Rico. We found that a multidisciplinary Managed Medicare chronic disease management program in Puerto Rico targeting enrollees with DM and CKD Stages 1–3, followed from 6 months to 2 years, could prevent or delay the reported relentless progression of CKD in many patients. We also found that compared to historical controls, the CDM program was possibly more effective in stabilizing or improving CKD status in patients with CKD Stage 3. This highlights the potential of proactive, multidisciplinary team-based interventions for stabilization and regression of CKD Stages 1–3 for many patients, even in populations facing significant structural barriers. Randomized pragmatic trials of the CDM program to inform decisions [31,32], as well as longer-term follow-up on kidney function and clinical outcomes in this high-risk population, are warranted.

## Figures and Tables

**Table 1:** Baseline characteristics by CKD stage, N = 2095.

	Overall N (%)	Stage 1 CKD N (%)	Stage 2 CKD N (%)	Stage 3 CKD N (%)
Gender				
Male	857 (40.9)	281 (40.1)	306 (42.0)	270 (40.6)
Female	1238 (59.1)	420 (59.9)	423 (58.0)	395 (59.4)
Age (years)				
27–64	274 (13.1)	145 (20.7)	99 (13.6)	30 (4.5)
65–74	808 (38.6)	283 (40.4)	323 (44.3)	202 (30.4)
75–84	770 (36.8)	219 (31.2)	253 (34.7)	298 (44.8)
85+	243 (11.6)	54 (7.7)	54 (7.4)	135 (20.3)
Diabetes, n (%)	586 (28.0)	203 (28.6)	185 (25.4)	198 (29.8)
Hypertension, n (%)	301 (14.4)	92 (13.1)	106 (14.5)	103 (15.5)
	Mean ± SD	Mean ± SD	Mean ± SD	Mean ± SD
eGFR (mL/min/1.73 m^2^)	71 ± 19	91 ± 3	73 ± 8	48 ± 8
BMI (kg/m^2^)	28.9 ± 6.0	29.2 ± 6.3	29.1 ± 6.0	28.6 ± 5.6
Systolic BP (mmHg)	127 ± 14	126 ± 14	127 ± 14	128 ± 14
Diastolic BP (mmHg)	75 ± 8	75 ± 8	75 ± 8	74 ± 8
Hemoglobin A1c (%)	6.9 ± 3.3	7.0 ± 3.7	6.8 ± 4.0	6.8 ± 1.5
LDL (mg/dL)	92.8 ± 34.5	94.8 ± 35.1	93.7 ± 34.2	89.5 ± 33.9
Medications, n (%)				
ACEi	21 (1.1)	7 (1.2)	6 (0.9)	8 (1.3)
ARB	131 (6.9)	39 (6.5)	44 (6.4)	48 (7.9)
SGLT2/GLP1	136 (7.2)	36 (6.0)	32 (4.7)	68 (11.2)

eGFR = estimated glomerular filtration rate; CKD = chronic kidney disease; BMI = body mass index; BP = blood pressure; LDL = low-density lipoprotein; ACEi = angiotensin-converting enzyme inhibitor; ARB = angiotensin receptor blocker; SGLT2 = sodium–glucose co-transporter; GLP1 = glucagon-like peptide. Diabetes and hypertension numbers are less than total due to missing values.

**Table 2:** Baseline Participant Characteristics Stratified by Stable CKD versus CKD Progression.

	CKD Stage Stable or Regressed * N (%)	CKD Stage Progressed ** N (%)
Gender		
Male	697 (42.2)	160 (36.0)
Female	953 (57.8)	285 (64.0)
Age (years)		
27–64	225 (13.6)	49 (11.0)
65–74	624 (37.8)	184 (41.4)
75–84	604 (36.6)	166 (37.3)
85+	197 (11.9)	46 (10.3)
Diabetes, n (%)	452 (27.4)	134 (30.1)
Hypertension, n (%)	233 (14.1)	68 (15.3)
	Mean ± SD	Mean ± SD
eGFR (mL/min/1.73 m^2^)	68.6 ± 18.5	81.8 ± 15.9
BMI (kg/m^2^)	28.9 ± 6.0	29.1 ± 5.8
Systolic BP (mmHg)	127 ± 14	127 ± 15
Diastolic BP (mmHg)	75 ± 8	75 ± 8
Hemoglobin A1c (%)	6.9 ± 3.6	6.8 ± 1.4
LDL (mg/dL)	92.5 ± 34.2	93.6 ± 35.4
Medications, n (%)		
ACEi	15 (0.99)	6 (1.5)
ARB	97 (6.4)	34 (8.7)
SGLT2/GLP1	110 (7.3)	26 (6.7)

* Patients with CKD Stage Stable or Regressed n = 1650 (78.8%). ** Patients with CKD Stage progressed n = 445 (21.2%). eGFR = estimated glomerular filtration rate; CKD = chronic kidney disease; BMI = body mass index; BP = blood pressure; LDL = low density lipoprotein; ACEi = angiotensin-converting enzyme inhibitor; ARB = angiotensin receptor blocker; SGLT2 = sodium–glucose co-transporter; GLP1 = glucagon-like peptide.

**Table 3:** CKD stage at end of follow-up period (6–24 months).

	Remained at Stage N (%)	Regressed to a Less Severe Stage N (%)	Progressed to a More Severe Stage N (%)
CKD Stage 1	392 (55.9)	N/A	309 (44.1)
CKD Stage 2	501 (68.7)	118 (16.2)	110 (15.1)
CKD Stage 3	504 (75.8)	135 (20.3)	26 (3.9)

CKD = chronic kidney disease.

## Data Availability

Data is unavailable to the public due to privacy restrictions.
